# Microwave assisted green synthesis of silver nanoparticles using *Trigonella Hamosa L.*plant extract for the photodegradation of some water pollutants

**DOI:** 10.1038/s41598-025-21112-4

**Published:** 2025-10-27

**Authors:** M. Nageeb Rashed, Eman Abdelrady, Tereasa M. Ghabrial

**Affiliations:** 1 Department of Chemistry, Faculty of Science, Aswan University, Aswan, Egypt; 2 Unit of Environmental Studies and Development, Aswan University, Aswan, Egypt

**Keywords:** *Trigonella hamosa* L., Silver nanoparticles, Microwave, Photodegradation, MB dye, Paracetamol, Biotechnology, Chemistry, Environmental sciences, Materials science, Nanoscience and technology

## Abstract

Biological techniques are regarded as more sustainable to synthesize nanoparticles compared to physical and chemical ones due to their environmentally friendly characteristics and cost efficiency. In this research, silver nanoparticles (AgNPs) were synthesized using aqueous extracts of *Trigonella hamosa Leave* via conventional and microwave-assisted methods. The leaf extract of *Trigonella hamosa *L. was effectively employed as both a reducing and stabilizing agent in the preparation of AgNPs. Microwave-assisted synthesis of AgNPs shows average size of crystal (14 nm) less than that obtained without microwave (16 nm). The prepared AgNPs was characterized using XRD, FTIR, HR-TEM, and UV–vis. The UV–visible spectrophotometer identified the formation of AgNPs by observing the surface plasmon resonance absorption (SPR) band peak at approximately 430 nm. The HR-TEM and XRD studies show that the AgNPs particles have an average diameter of 14 nm and are nearly spherical, as indicated by the TEM. Synthesized AgNPs have been successfully used as a catalyst for photodegradation of methylene blue dye (MB) and paracetamol under sunlight and/or visible lamp irradiation. The MB dye experienced degradation rates of 96.2 and 94.9%, whereas paracetamol showed degradation percentages of 94.5 and 92% when subjected to sunlight and visible lamp irradiation, respectively. The results of this research indicate that AgNPs may serve as a viable alternative for the effective and safe elimination of hazardous organic pollutants.

## Introduction

Nanotechnology is presented as a swiftly advancing area of contemporary research, focusing on the creation of materials at the nanoscale, which generally ranges from 1 to 100 nm^1^. Metal nanoparticles (NPs) possess a distinctive volume-to-surface ratio along with unique physical and chemical characteristics, allowing them to be utilized in various areas such as water purification and catalysis^2^, pharmaceutical and medicine industries^3,4^, optics^2^, cosmetics, biomedicine ^5^, chemical factories, drug delivery, edible products, electron transistors, mechanics, and electrochemical devices^6,7^, and cancer analysis and treatment^8^. Furthermore, there is an increasing interest in creating environmentally friendly methods for NP synthesis that utilize biocompatible substances instead of expensive and dangerous chemicals^9^.

Nanoparticles (NPs) are synthesized through several techniques, including physical chemical and biological processes. Physical and chemical methods seem to be very expensive, hazardous, lengthy, involve hazardous chemicals, and may pose risks to the environment. The physical techniques consist of several methods such as evaporation, condensation, laser ablation, ultraviolet microwave radiation, sono-electrochemical, and photochemical^10^. Chemical processes include spray pyrolysis, metal salt precursor reduction in solution, micro-emulsions, sonochemical, and microwave-assisted^11^. Biological processes appear to be straightforward, quick, non-harmful, reliable, and environmentally friendly methods that can yield precise sizes and shapes when conditions are optimized^12^.

The green synthesis of NPs is a special method since it just needs plants for the process, which is a good substitute due to its cost-effectiveness, environmental sustainability, and ease of expansion for large-scale processing. High temperatures, pressures, and toxic chemicals are not necessary when using the green method, which operates under mild conditions. Some different phytoconstituents, such as alkaloids, terpenoids, and flavonoids are used as reducing agents for synthesis of AgNPs^13^. Therefore, it’s highly recommended for synthesis of NPs based on the development of a biologically inspired procedure^14,15^.

Metal nanoparticles, particularly AgNPs, have gained interest due to their strong surface plasmon resonance (SPR) effect, which enhances photocatalytic activity under light irradiation. AgNPs can be found in different geometries, including hexagonal plates, decahedral nanoparticles, pentagonal nanorods, nanowires, nanocubes, nanoflowers, and nanospheres^16^. They can exist as nanocapsules, nanorods, carbon nanotubes, quantum dots, nanoemulsions, and fullerenes^17^. These AgNPs are characterized by their conductivity, catalysis, chemical stability, anti-fungal, antimicrobial, anti-viral, and anti-inflammatory potential^18,19^. They have been integrated into products like fibers, cosmetic goods, superconducting materials^20^, food containers, paints, textile products, dressings, catheters, disinfection sprays^11^, shampoos, detergents, shoes, toothpaste, and medications^21^.

In recent years, biological entities such as plants, bacteria, fungi, hormones^22^, proteins^23,24^, and urease^25^ have been utilized in the NPs synthesis. A one-protein technique could be used to quickly prepare biomolecule-functionalized NPs at room temperature. These protein-functionalized nanoparticles were successfully produced for various proteins with differing molecular weights and isoelectric points.

The synthesis of green plant-based materials not only lowers preparation costs but also eliminates the need for specialized techniques and plant preparations for creating nano-composites, as they utilize readily available local plant seeds^26^. The synthesis of NPs facilitated by plants is increasingly favored because of its benefits, including being non-toxic, environmentally friendly, and more economical than methods involving microorganisms that need sterile conditions for cell culture^27^.

There are 11 annual herbaceous species in the genus *Trigonella*, which is a member of the *Fabaceae* family, in the Egyptian flora. One of these florae is *Trigonella hamosa*, which is an annual herb that is common in Egypt^28^. Many phytochemistry studies of *Trigonella hamosa* have been carried out. According to Hamed^29^ steroidal saponins were isolated from the Egyptian *Trigonella hamosa* seeds and showed biological activities against diabetes, lipid profiles, and glucose homeostasis^30^. The isolated saponins from *Trigonella hamosa* seeds mitigated the harmful effects of chronic diabetes^31^.

*Trigonella hamosa* L. leaves extract was utilized in our study as a stabilizing and reduction agent in the preparation of AgNPs. *Trigonella hamosa *L. leaves extract, which is used as a reducing agent, not only offers numerous advantages but also requires fewer chemicals. Using microwave assistance in the synthesis of AgNPs makes up a more effective and eco-friendly method. Various pharmaceutical pollutants have been identified in wastewater^32^, surface and groundwater^33^, and drinking water^34^. Specifically, compounds like antibiotics, hormones, and analgesics are commonly found in the aquatic environment^35^.

The existence of pharmaceutical pollutants in drinking water is considered as a significant problem because they can lead to carcinogenic disorders, which have a negative impact on human health and cause public concern these substances have harmful effects such as resistance to pathogenic microorganisms, toxicity, and endocrine^34^.

Acetaminophen, often known as paracetamol, is a common, highly water-soluble, non-biodegradable substance that is found in over 100 prescription medicinesb^36^. Paracetamol (PCA) is one of the most common drugs which used for treating a variety of colds, influenza, and headaches.

Dyes are aromatic organic compounds described as colored, and ionizing, they are used in the textile industry^37^. Organic dyes are becoming more and more common in daily life as a result of increasing population, civilizations, and consumerism. Organic dyes are used in different sectors such as food, plastics, leather, paints, cosmetics, paper, textiles, and medicines. These non-biodegradable materials are a serious environmental hazard that has to be eliminated from the ecosystem. Both the environment and human health are harmed by these synthetic organic dyes.

The management of organic dyes that reach water bodies through wastewater is considered the most difficult problem in the postproduction of products from these sectors.

Coagulation, reverse osmosis, adsorption, and filtration have all been used in traditional color removal techniques. These hues have aromatic structural stability, so their removal from water is very difficult. Kumari, et al.^38^ studied the removal of methylene blue from polluted water using composite hydrogel based on pine needles, chitosan, and gelatin as adsorbent.

Many techniques, including electrocoagulation, flocculation, UV light degradation, activated carbon sorption, and redox treatments, are regularly used for reducing dyes^39,40^. One of the most promising substances for reducing synthetic dyes is nanocatalysts.

Recently, various methods, such as electrochemical^41^, ozonation^42^, Fenton and photo-Fenton^43^, and oxidation techniques H_2_O_2_/UV^44^ have been developed. Heterogeneous photocatalysis is among the most promising advanced oxidation techniques (AOPs) for extracting paracetamol from a water medium.

There have been reports about the photocatalytic degradation of methylene blue by green-synthesized AgNPs under sunlight. Exposure to sun radiation (exposure duration) has a vital role in photocatalytic degradation activity of AgNPs. Through the photocatalytic degradation of MB dye under sunlight, the dye color was dark blue and changed to light blue/colorless after reaction time. With a UV–visible spectrometer, the absorbance can be measured to determine that the dye’s absorption intensity decreases with exposure time, while in the presence of AgNPs catalyst, the dye degradation is shown by a decrease in the absorption intensity, which also indicates a reduction in the dye. The rate of AgNPs degradation depends on many factors, such as the size of the particles, morphology, structure, and pollutant concentration. AgNPs size is the most important factor in dye degradation, as the rate of reaction (dye degradation) increases with decreasing particle size, and also, because smaller particles have a larger volume -to- surface ratio and so they adsorb more strongly to the reactants. According to reports, in the dye degradation process, the AgNPs are the mediators of transferring the electrons. Additionally, it has been suggested that AgNPs’ surface plasmon resonance (SPR) may be excited, which could explain photocatalytic activity. In other words, the photonic excitation of AgNPs speeds up the dye degradation rate^45^.

Several researches have been published about the photocatalytic activity of green AgNPs on MB dye. According to Saha, et al.^46^ reported the complete degradation of MB dye in 10 min in the presence of 3.0 mL of Ag nanoparticles with 17 nm average particle size. Additionally, Banu, et al.^47^ syntheses AgNPs particle with average size 15 nm using Bael Gum as a stabilizer and reluctant and applied it for degradation of MB dye using microwave assitance. Also, Kuyyogsuy, et al.^48^ reported 90% degradation of methylene blue in 90 min with an average 48 nm particle size.

It has been found that the AgNPs size has an important effect on the degradation of different colors. The smaller the silver particle, the faster it degrades and the more effective the degradation is. The degradation is faster and more efficient when the silver particle is smaller. Additionally, efficacy and dye degradation are influenced by silver nanoparticle concentration, the higher the concentration, the greater the dye degradation.

Although there have been several research studies on the preparation of AgNPs from plant extracts to the best of our knowledge, there is no published literature on the preparation of AgNPs using *Trigonella hamosa* L. plant. So far, the objectives of this study are to:- (i) prepare AgNPs using *Trigonella hamosa* L. for the first time. (ii) Compare the preparation methods using microwave irradiation. (iii) photodegradation of methylene blue dye and paracetamol drug using the prepared AgNPs under sunlight and visible lamp irradiation.

## Materials and methods

### Materials and reagents

#### Plant material collections

**Fig. 1:**
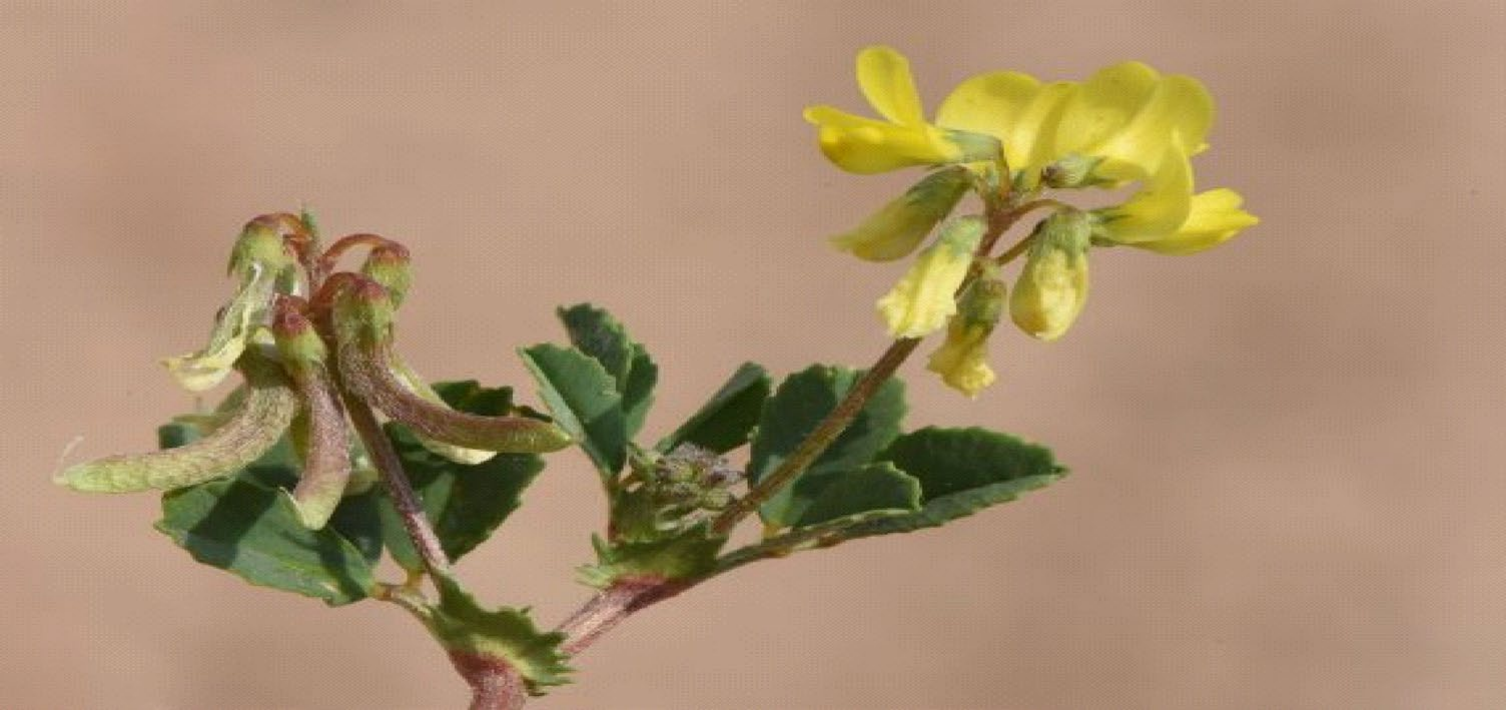
Picture of *Trigonella hamosa* plant.

The aerial parts of *Trigonella hamosa* L. plant (Fig. 1) were collected from Botanical Garden at Aswan University, Aswan Government, Egypt after permission of Aswan University Garden sector. The collected plant material was established in compliance with the national guidelines. They were kindly authenticated by Prof.Mohamed Gaber Shadeed (Department of Botany, Faculty of Science, Aswan University). A voucher specimen no. 012001 was deposited in the Herbarium of Botany Department, Faculty of Science, Aswan University.

#### Reagents, chemicals and instruments

Analytical grade chemicals, silver nitrate (1 mM AgNO_3_), methylene blue dye, and paracetamol were procured from Sigma-Aldrich Co.Domestic microwave oven (ELEKTA) working at a power of 700 W and frequency of 2450 MHz was used for predation of AgNPs. Visible lamp (1000 W) was used as an irradiation source. A TOMOS UV spectrophotometer was used to perform UV–vis spectrum analysis. The FTIR spectrum was recorded on a CLASS LASER PRODUCT-4600 spectrometer with ATR attachment. A Bruker co D8, Germany X-ray spectrometer was used for XRD measurement. High-resolution transmission electron microscopic (HR-TEM) images were taken using a JEOL JEM-2100 microscope.

### Methods

#### Preparation of *T. hamosa* L. leaf extract

**Fig. 2:**
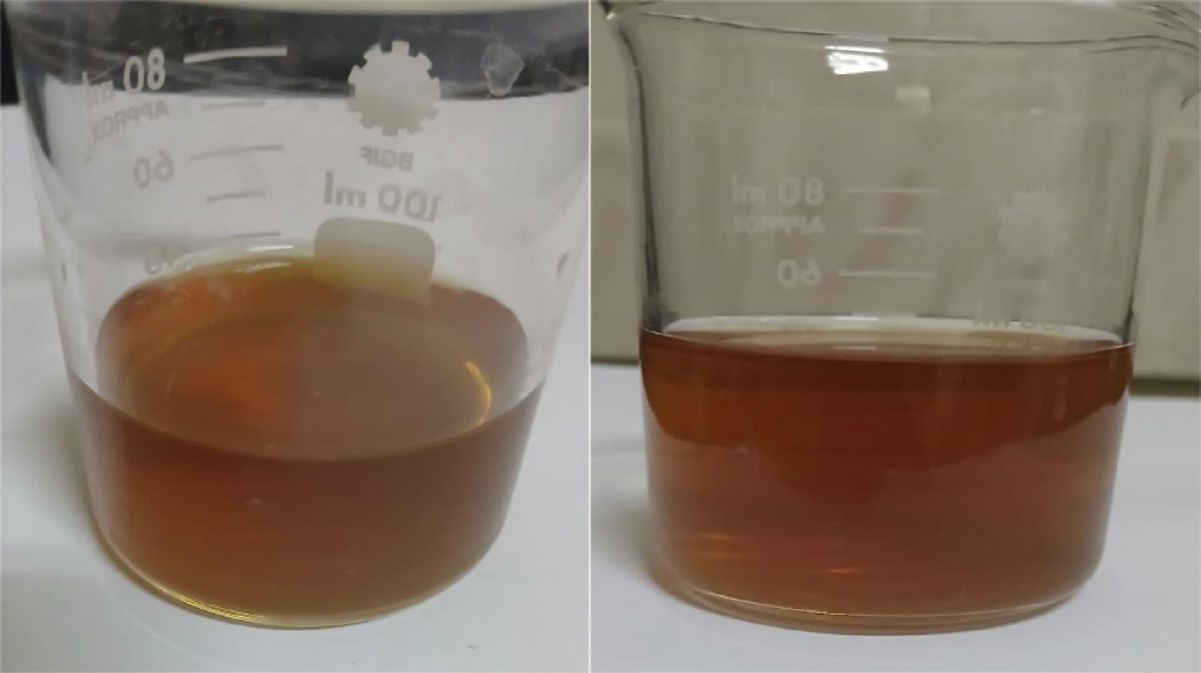
Picture of *Trigonella hamosa *L. leaves extract.

The leaves of *T. hamosa *L. were washed thoroughly in tap water and then in distilled water, and they were laid out evenly and allowed to air dry. The dried fresh leaves (10 g) were cut into thin strips, placed into a 200 mL glass flask, boiled with bidistilled water at 60 °C in 1 h, cooled, and filtered with Whatman filter paper using a vacuum filter. The *Trigonella hamosa* L. leaf extract had a light brown color (Fig. 2). For later usage, the extracted solution was kept in a refrigerator.

#### Synthesis of AgNPs

##### Microwave assisted synthesis of AgNPs

A 250 mL beaker containing 90 mL of 1 mM silver nitrate solution was used for microwave-assisted synthesis. 10 mL of *T. hamosa *L. leaf extract was added to the contents and stir well at pH 6.9. After then the beaker with its contents was put in a domestic microwave oven (ELEKTA) that ran at 700 W and 2450 MHz, and it was exposed to microwave radiation for five minutes. After the radiation time, AgNPs solution was centrifuged at a speed of 10,000 rpm for 10 min for separation of the green-synthesized AgNPs. The resultant pellets were washed three times with deionized water and let at room temperature to dry in order to get rid of the contaminants that had settled down with the synthesized NPs. After five minutes of microwave irradiation in the 300–700 nm region, the reaction mixture was analyzed using UV–vis spectrometer to observe the formation of silver AgNPs.

##### Non microwave assisted synthesis of AgNPs

In order to understand how microwave heating affects the rate of AgNPs formation, the synthesis of AgNPs was also carried out without use of microwave. For that, 90 mL AgNO_3_ aqueous solution (1 mM) was added to 10 mL of *T.* *hamosa* L. extract at pH 6.9, heated at 70 °C with stirring for 3–4 h.

To investigate the AgNPs formation, the reaction mixture was regularly analyzed using UV Vis spectroscopy.

#### Photocatalytic degradation of MB dye using AgNPs

Typically, a stock solution was made by adding 10 ml of MB dye to 1000 mL of bidistilled water. About 100 mL of methylene blue dye solution with a concentration of 10 ppm was mixed with 10 mg of synthesized AgNPs at pH = 6.5. Additionally, a control sample was kept in the absence of AgNPs. To ensure that the working solution was in equilibrium, the reaction suspension was thoroughly mixed by magnetic stirring for 30 min prior to exposure to radiation. The dispersion was then placed under sunlight and observed for 24 h, from sunrise to sunset. Aliquots of a 2–3 mL suspension were filtered at set time intervals and used to determine the dye’s photodegradation. A UV–Vis spectrophotometer was used to measure the absorbance spectra of the supernatant at different wavelengths. The absorbance value at 660 nm was used to determine the dye concentration throughout degradation. All studies were carried out in a comparable photoreactor. The reactor was put under a visible lamp for 7 h at room temperature (24 ± 2 °C). A UV–Vis spectrophotometer was used to measure the absorbance spectra of the supernatant at different wavelengths. The absorbance value at 660 nm is used to calculate the dye concentration during degradation.

The percentage of MB degradation (DE_MB_) was obtained by the following Eq. (1):

1 $$\% DE_{MB} = \left( {C_{o} - C} \right)/C_{o} * 100$$

where C₀ and C are the initial concentration of MB and the final concentration, respectively.

#### Photocatalytic degradation of Paracetamol (PCA) using AgNPs

The degradation of paracetamol was used to evaluate the photocatalytic degradation efficiency of AgNPs. A stock paracetamol solution was made by dissolving 0.5 g of paracetamol in 0.5 L bidistilled water. The working paracetamol solution 50 ppm was prepared by diluting the stock one the resulting stock solution was then diluted to prepare a working solution. Photodecatalytic degradation tests were carried out using a photoreactor (200 mL) equipped with a magnetic stirrer and a visible lamp. Every catalytic test was carried out at room temperature (24 °C) using visible lamp illumination. For sunlight irradiation, all experiments were conducted in the same photoreactor but under sunlight irradiation. The reactor was exposed to direct sunlight for 24 h, from morning until sunset on sunny days (temperature was 38 ± 2 °C) with constant stirring. Aliquot degraded samples were taken at a specific time period. The resulted Paracetamol concentration was monitored using a UV–vis spectrophotometer at 325 nm. The paracetamol percentage degradation was calculated using Eq. (2).

2 $${\mathrm{PCA}}\;{\mathrm{Degradation}}\% = \left( {C_{o} - C} \right)/C_{o} * 100$$

where C_0_ is the initial paracetamol concentration, C is the paracetamol concentration in the reactor after illumination.

#### Characterization

Ultraviolet–visible spectral analysis was performed utilizing a TOMOS UV spectrophotometer. FTIR spectrum was obtained using a CLASS LASER PRODUCT-4600 spectrometer equipped with an ATR attachment. XRD measurements were conducted on a Bruker co D8 spectrometer from Germany. HR-TEM images were obtained with microscope JEOL JEM-2100.

## Results and discussion

### UV–vis spectroscopic

**Fig. 3:**
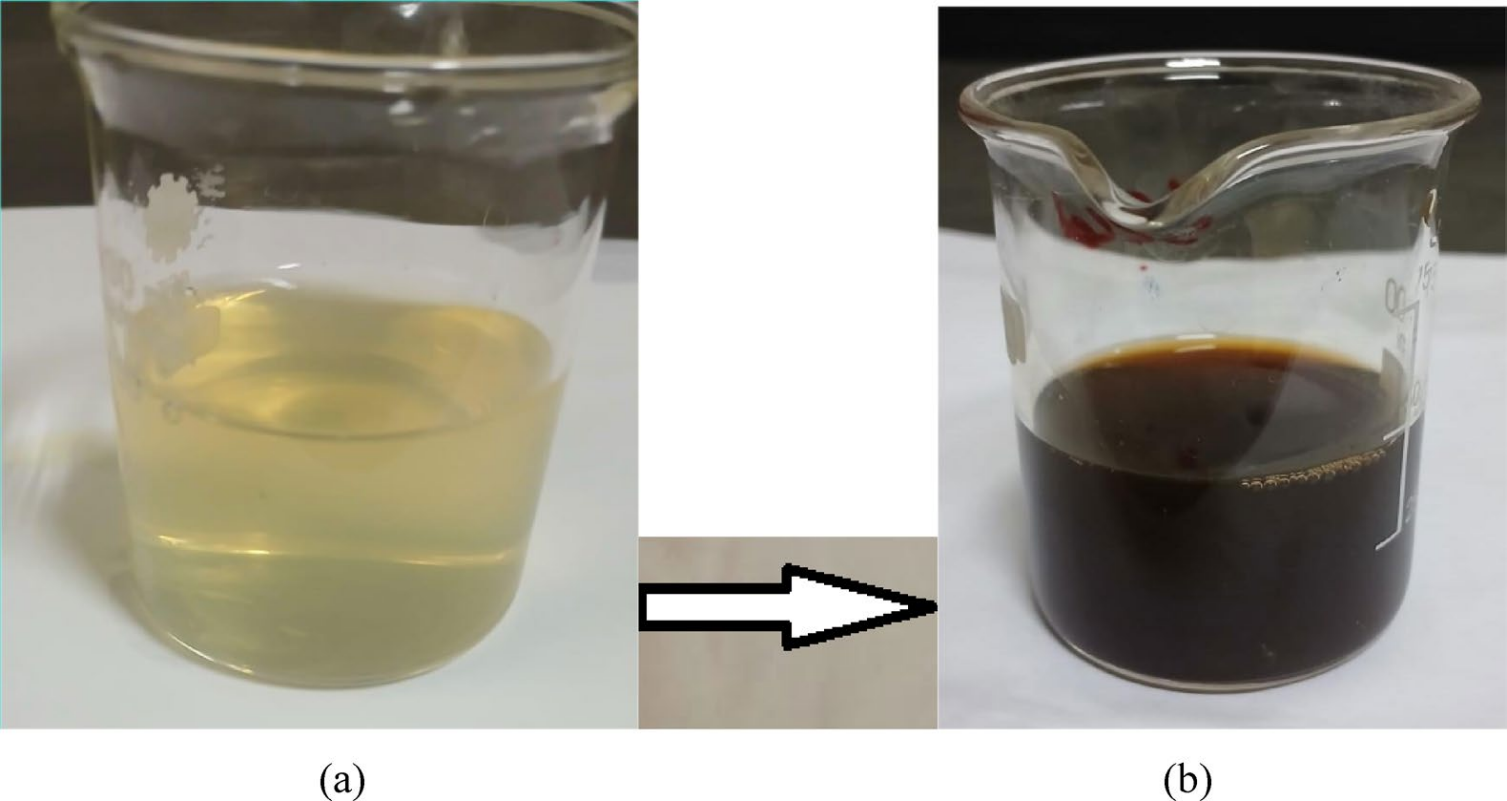
Images of (**a**) mixture of plant extract and AgNO_3_ before microwave and (**b**) AgNP-after microwave.

Ultraviolet–visible spectroscopy is recognized as a method utilized to verify the formation and stability of metal nanoparticles, as the solution displays an absorption band within the UV–vis range due to the surface plasmon oscillations of metal electrons, which provide insights into the shape and size of the nanoparticles. The mixture of AgNO_3_ and T. *hamosa *L. plant extract appeared pale yellow; the solution’s color shifted to a yellowish brown when subjected to microwave radiation. (Fig. 3).

**Fig. 4:**
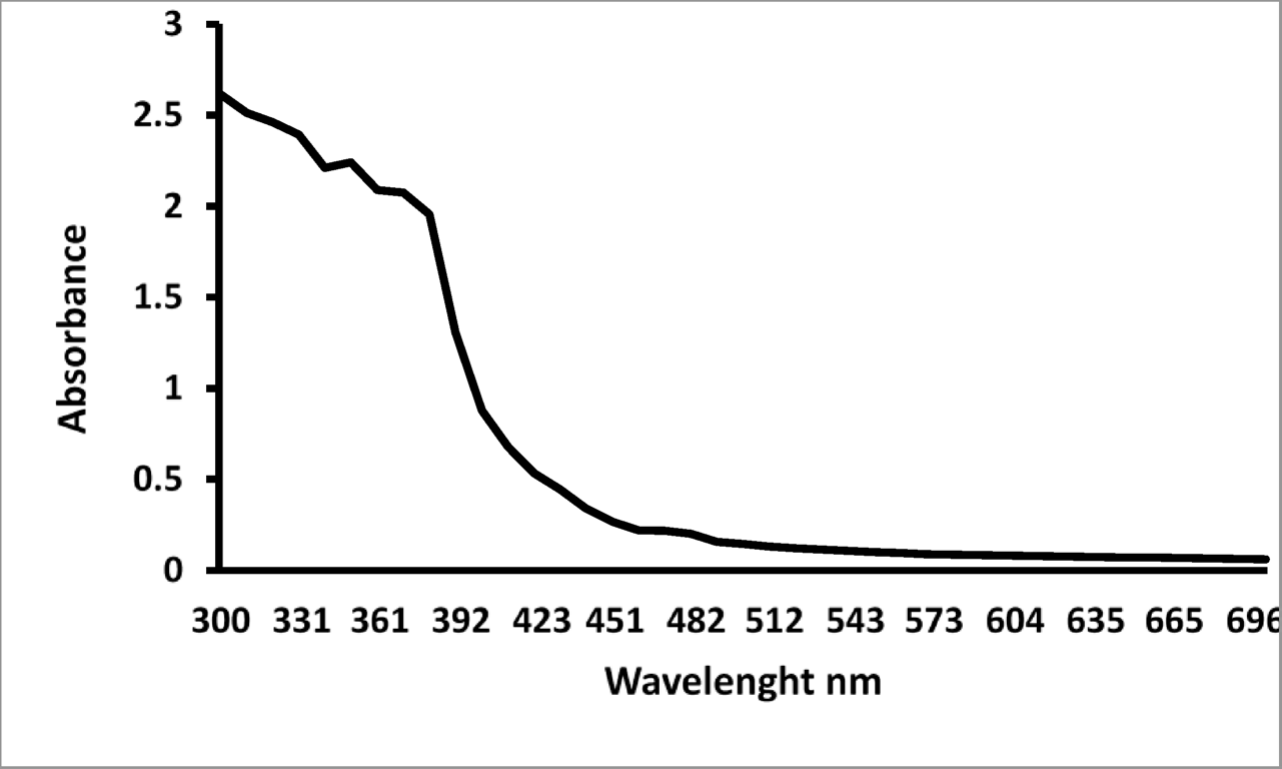
UV–vis absorption spectra of T.hamosa L leaves extract.

There was no peak between 300 and 700 nm for *T. hamosa *L. plant extract before microwave irradiation (Fig. 4).

**Fig. 5:**
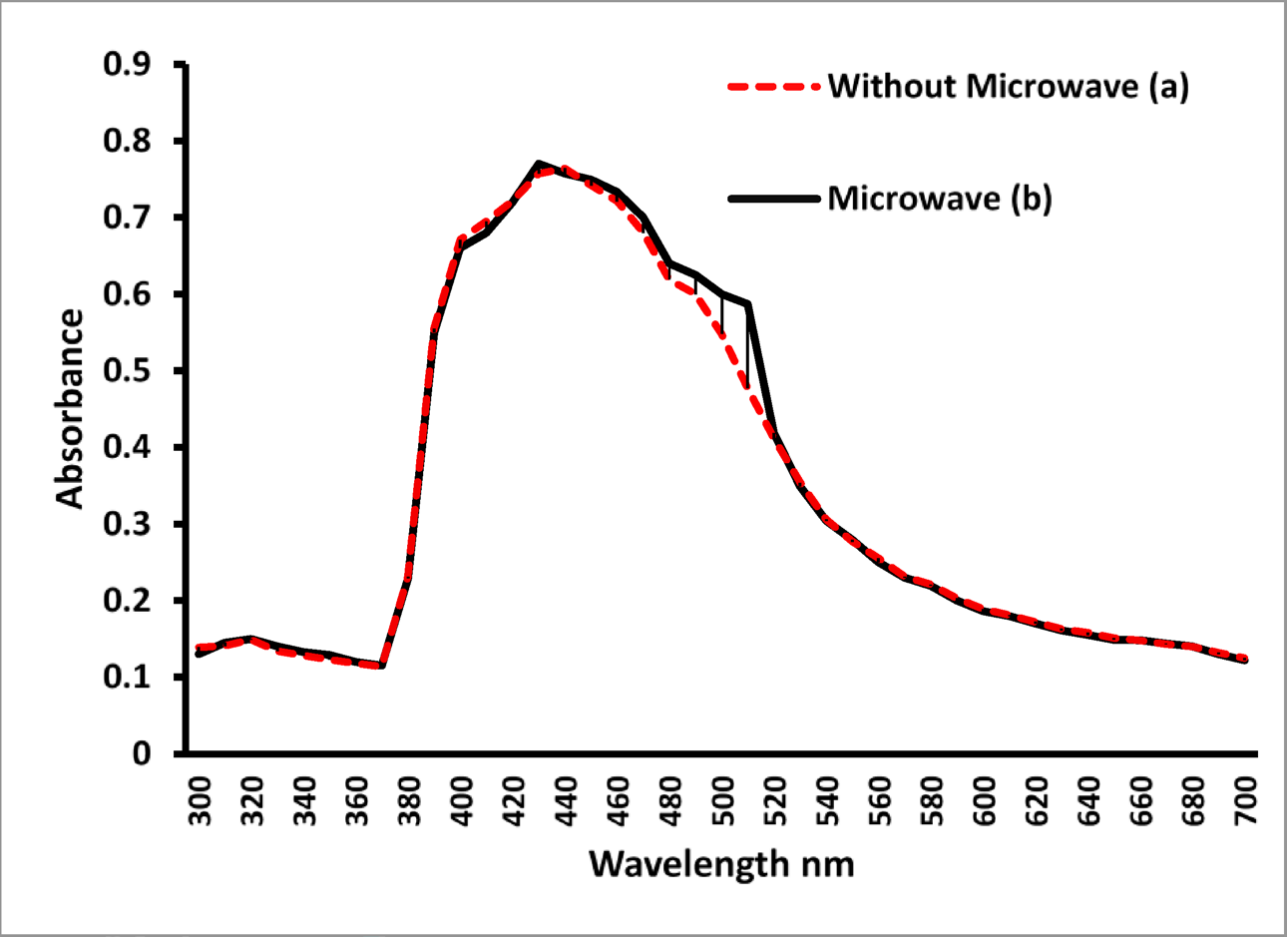
UV–vis absorption spectra of AgNP synthesized with T.hamosa L leaves extract, a) without microwave irradiation (60 min stirring time, 70°C), b) under microwave irradiation after 5 min.

AgNPs were prepared using *T. hamosa *L. leave extract without microwave irradiation to investigate the impact of microwave irradiation on the formation rate of AgNPs. The UV–vis.spectra of the resulted reaction solution without microwave irradiation was recorded at 60 min intervals (Fig. 5a). After 1 h reaction time, a peak was observed at 440 nm due to surface plasmon resonance (SPR) of AgNPs.

The microwave method is a faster way to synthesize nanoparticles since it reduced Ag⁺ ions into Ag and simultaneously formed stable nanoparticles in about 5 min. Figure 5b illustrates the UV–Vis spectra collected during the microwave synthesis of AgNPs. At 5 min of the irradiation process, a peak was detected at 430 nm, attributed to the surface plasmon resonances (SPR) of AgNPs; this peak’s intensity grew with the duration of irradiation while its position remained relatively stable. After five minutes of exposure, there was no further increase in absorbance, indicating that all Ag⁺ had been converted to Ag. The SPR band emerges due to the collective oscillations of the conduction electrons in the nanoparticles and is significantly affected by the size and shape of the nanoparticles^49^. It has been noted that the SPR band shifts towards longer wavelengths as the particle size increases (14–16 nm). In this synthetic approach, the microwave-assisted synthesis produces smaller nanoparticles in significantly shorter reaction times compared to traditional methods without microwave assistance. TEM analysis provides additional confirmation of this observation.

Anis, et al.^27^ reported the presence of an absorption band in the range of 400–500 nm when synthesized AgNPs using pineapple leaves waste mediated by microwave-assisted extraction.

Microwave synthesis has been utilized for nanoparticle production due to its ability to deliver quick and uniform heating of the starting materials. The penetration ability of microwave irradiation allows for even heating of the reaction mixture. This leads to rapid growth of crystals, resulting in crystallites with a narrow size distribution. In contrast to traditional methods, microwave irradiation synthesis offers the benefit of shorter reaction times, which can be attributed to the combined electric and magnetic forces of the microwave that generate friction and molecular collisions.

*T. hamosa *L. leaf extract is abundant in phenolic compounds and flavonoids, among other phytochemicals. These phytochemicals serve as both stabilizing and reducing agents during the production of AgNPs. By adding the leaf extract to the AgNO_3_ solution, the silver ions are reduced to silver atoms, which then aggregate to form AgNPs. Also, the phytochemicals coat the nanoparticles, preventing them from coalescing^50^. The position of the AgNPs SPR band remains unchanged even after being stored at room temperature for several weeks, as demonstrated by UV–vis spectra, indicating that the phytochemicals effectively stabilize the AgNPs.

### FTIR

The different functional groups in the *T. hamosa *L. leaf extract and on the surface of their synthesized AgNPs were identified by FTIR analysis. These functional groups have an important role in reducing Ag⁺ to Ag and stabilizing the synthesized AgNPs.

**Fig. 6:**
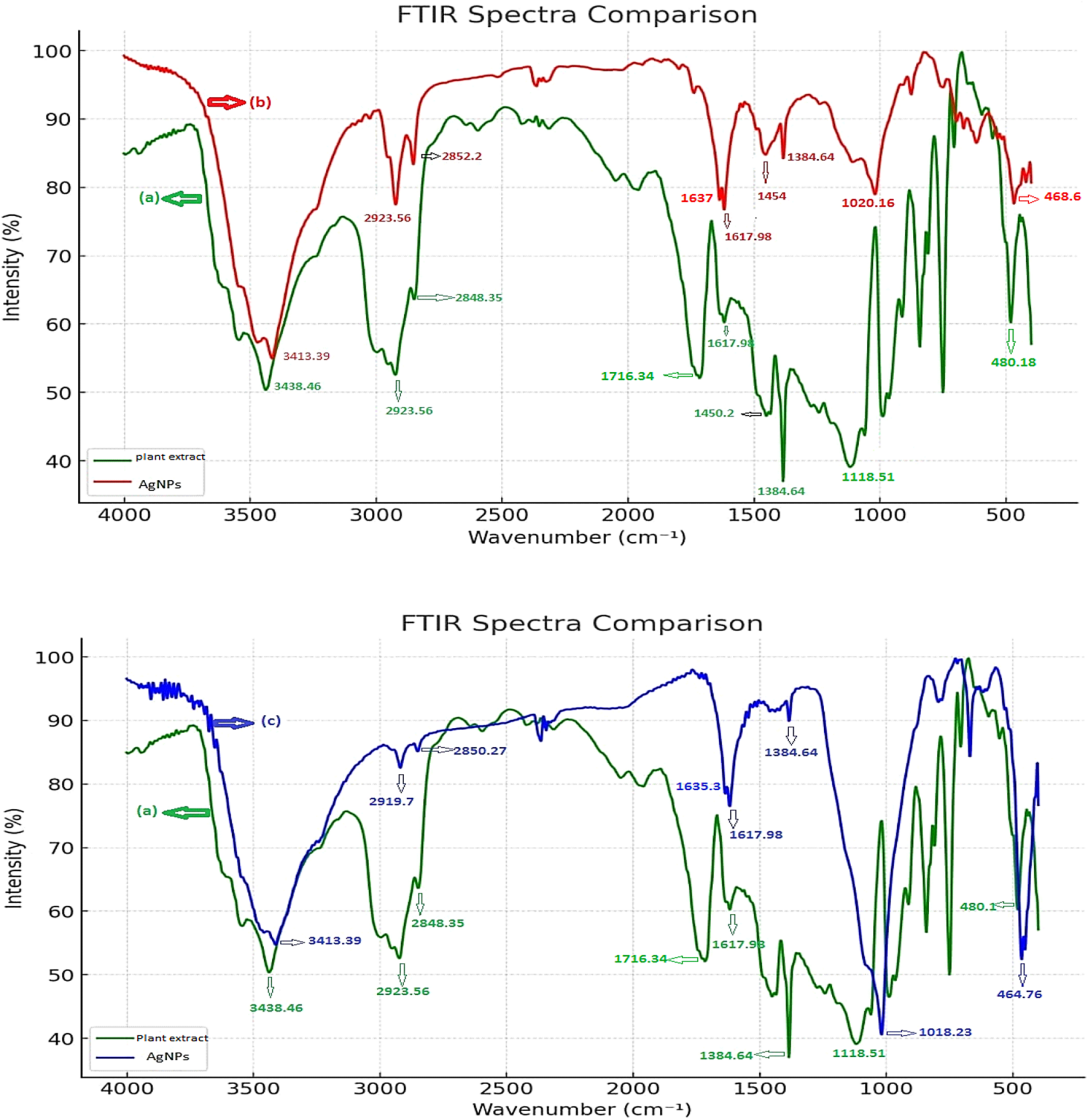
(**a**) FT-IR spectrum of an aqueous leaf extract, (**b**), and (**c**) synthesized AgNPs without and by microwave.

The impact of *T. hamosa* leaves extract in stabilizing the AgNPs was examined using FTIR spectroscopy. The spectrum FTIR of the *T. hamosa* leaf extract (Fig. 6a) revealed prominent absorption peaks at 1118, 1384, 1716, 2923, and 3438 cm^−1^. The strong broad band at 3438 cm^−1^ indicates the presence of -OH functional groups found in alcohols and phenolic substances. An aliphatic-CH stretching band is located at 2923 cm^−1^. Band amide I of the proteins extracted from *T. hamosa* leaves has also been recognized to show a band at 1716 cm^−1^. The geminal methyl has a band at 1384 cm^−1^, while the ether (C–O–C) bond has a weak band at 1118 cm^−1^. These functional groups provided evidence for the presence of phytochemical constituents^51^. According to, Lin, et al.^52^, the carbonyl groups from protein peptides and amino acid residues and the hydroxyl groups of alcohols have a great affinity for binding metals. This enables them to function as encapsulating agents and therefore prevents the agglomeration of nanoparticles.

The FTIR spectrum of the AgNPs synthesized without microwave (Fig. 3b) showed bands at 3413, 2923, 1637, 1384, and 1020 cm^−1^. The CO- (NH) group is represented by a band at 1637 cm^−1^, while the -OH group in phenols and alcohols is represented by a band at 3413 cm^−1^. AgNO_3_ reduction is to be the cause of a small decrease in band intensity from 3438 to 3413 cm^−1^ by a value of 25 cm^−1^.The absorption frequency shift indicates that the -OH group is involved in the reduction process. Also, the shift of a band from 1716 to 1637 cm^−1^ indicates that the CO-(NH) group is also involved in the reduction of AgNPs. Additionally, bands at 2923, 1384, and 1020 cm^−1^ represent aliphatic –CH stretching, geminal methyl, and ether linkage, respectively. The FTIR spectrum of the synthesized AgNPs using microwave (Fig. 3c) showed bands at 3413, 2919, 1635, 1384, and 1018 cm^−1^. The –OH in phenols and alcohols is represented by a band at 3413 cm^−1^, while the stretching vibration of the CO-(NH) group is represented by a band at 1635 cm^−1^. AgNO_3_ reduction is as the result of a small decrease in band intensity from 3438 to 3413 cm^−1^ by 25 cm^−1^; and the absorption frequency shift indicates that the -OH group is involved in the reduction process. The shift of a band from 1716 to 1635 cm^−1^ indicates that the CO–(NH) group is also involved in the reduction of AgNPs. Additionally, bands at 2919, 1384, and 1018 cm^−1^ represent aliphatic –CH stretching, geminal methyl, and ether linkage, respectively.

In conclusion FTIR data suggested that AgNPs produced by *T. hamosa* leaf extract are composed of proteins and metabolites as terpenoids with functional groups of alcohols, amines, ketones, aldehydes, and carboxylic acids.

### XRD study

**Fig. 7:**
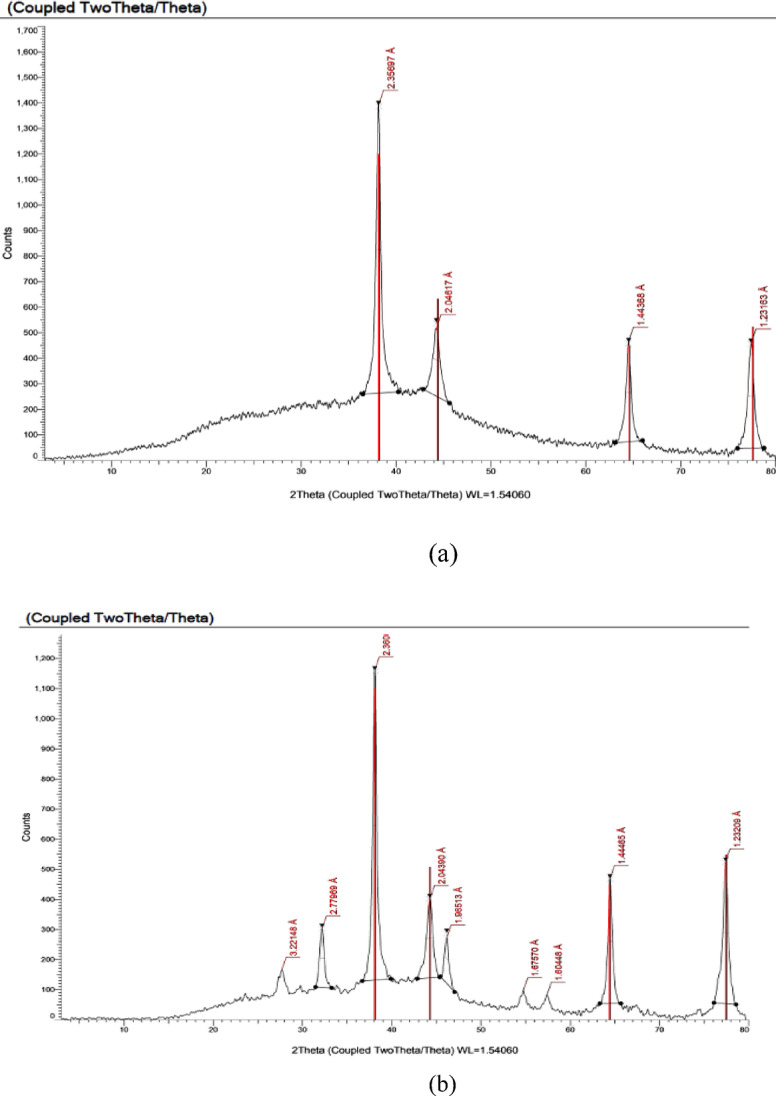
(**a**). XRD pattern of AgNP- *T. hamosa* prepared without microwave. (**b**) XRD pattern of AgNP- *T. hamosa* prepared using microwave.

The XRD analysis provides insights into the crystalline characteristics of the AgNPs. The XRD spectrum for AgNP-*T. hamosa* without microwave treatment (Fig. 7a) reveals diffraction peaks at 2θ angles of 38.139°, 44.26°, 64.51°, and 77.40°. In contrast, the spectrum obtained with microwave treatment (Fig. 7b) displays diffraction peaks at 2θ values of 32.17°, 38.098°, 44.26°, 46.14°, 64.45°, and 77.40°. The face-centered cubic structure of Ag allows these peaks to be attributed to the (111), (200), (220), and (311) crystallographic plane Joint Committee on Powder Diffraction Standard record (JCPDS File no. 04–0783) ^50^.

Based on the XRD results, AgNP-*T. hamosa* is predominantly crystalline. Debye–Scherrer’s formula is used to estimate the average size of the AgNPs^53^. The average of silver nanoparticles synthesized by *T. hamosa* without microwave is 14 nm, and the average of AgNPs synthesized by *T. hamosa* using microwave is 16 nm (Tables 1, 2). Table 3 shows published research about the particle size of AgNPs prepared by different plant extract.

**Table 1:** Crystallite size, FWHM, particle size and d-spacing values of AgNPs synthesized by *T. hamosa* extract without microwave with respect to 2θ.

Position 2θ (°)	FWHM 2θ (°)	d-spacing (Å)	Particle size (nm)
38.151°	0.379°	2.357 Å	15.7
44.229°	0.100°	2.046 Å	10.3
64.493°	0.351	1.443 Å	15.7
77.429°	0.369°	1.231 Å	14.0

Average particle size of the nanoparticles: 14.

**Table 2:** Crystallite size, FWHM, particle and d-spacing values of AgNPs synthesized by *T. hamosa* extract using microwave with respect to 2θ.

Position 2θ (°)	FWHM 2θ (°)	d-spacing (Å)	Particle size (nm)
32.174°	0.567°	2.779 Å	14.5
38.098°	0.461°	2.360 Å	18.1
44.260°	0.762°	2.044 Å	11.1
46.148°	0.491°	1.965 Å	17.5
64.457°	0.503°	1.444 Å	18.5
77.403°	0.650°	1.231 Å	15.5

Average particle size of the nanoparticles: 16.

**Table3:** Summary of the competitive research on MB dye degradation using different plants.

Plant	Size of AgNPs (nm)	% MB degradation	Condition	References
Fruit* Gmelina arborea*	17	100	10 min	Saha.et al., 2017
Cauliflower (*Brassica oleracea* var. *botrytis*)	35.08	97.57	150 min	Kadam et al.,2020
*Peltophorum pterocarpum* (leaf)	50	82	6 min	Adesetan, 2023
*Combretum indicum* (leaf)	48	90	90 min	Kuyyogsuy et al., 2021
Andographis Paniculata Leaves	85	84	16 h	Noorafshaet al., 2021
*Chlorella vulgaris*	55	96.51	3 h	Rajkumar et al., 2021
*Trigonella hamosa* L.	15	96.2	7 h	Our study using AgNPs synthesized with microwave

The AgNPs synthesized using *T. hamosa* leaf extract were optimized by different parameters including time, temperature, and pH. The results obtained and represented in Fig. 7a, b which show that the rate of AgNP nanoparticles formation is increased by increasing temperature. The particle size is initially reduced as a result of the reduction in aggregation of the growing nanoparticles. AgNPs formation is decreased in acidic conditions, but it is enhanced in basic one. At low pH values (pH 4), large nanoparticles were generated, while at high pH value (pH 9) small and highly dispersed AgNPs were obtained. At neutral pH, the normal AgNPs size is formed. The optimum time needed to complete the formation of AgNPs was 5 min using a microwave and 4 h without a microwave.

### HR-TEM study

**Fig. 8:**
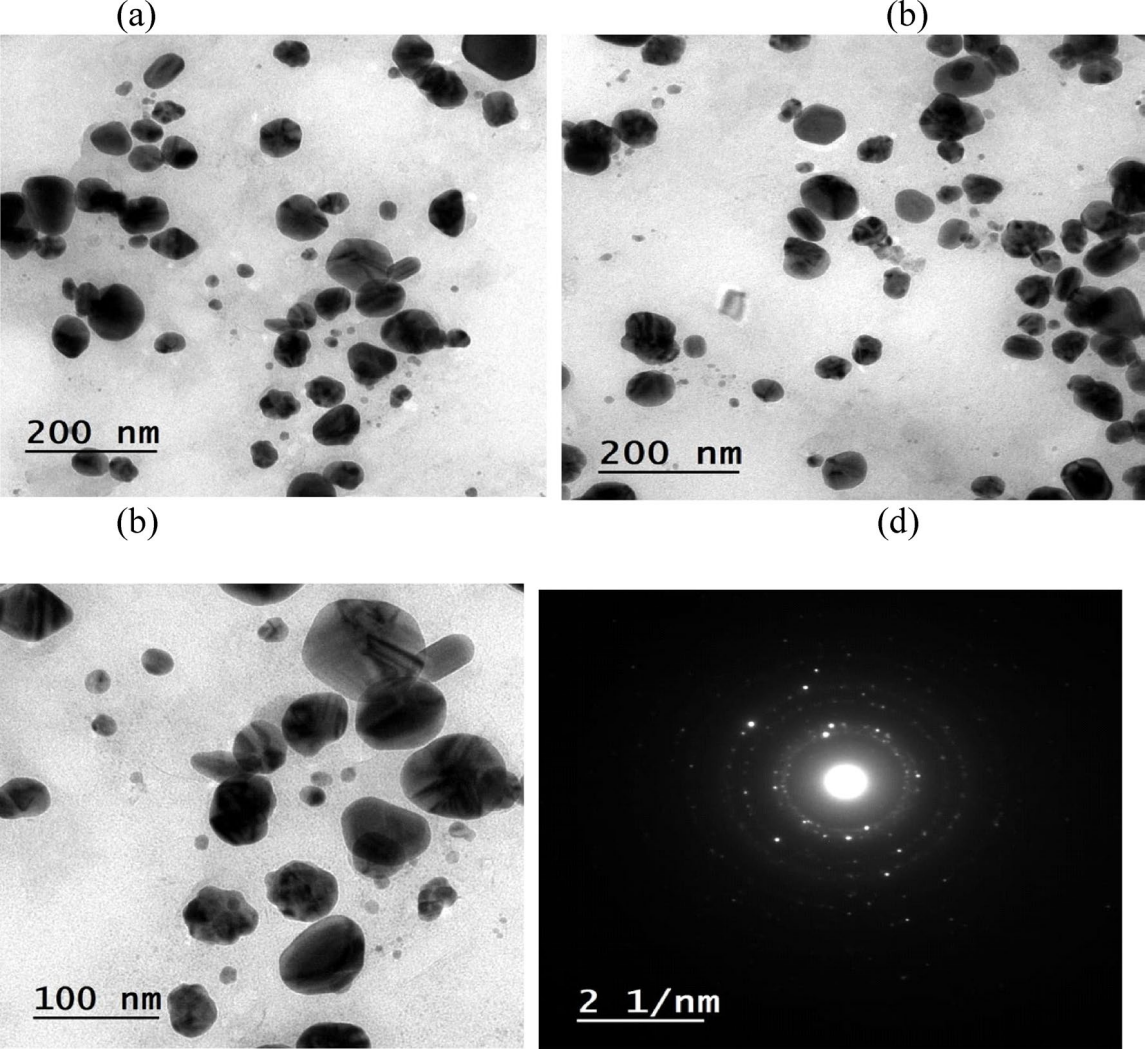
(**a**, **b** & **c**) TEM images of AgNP-synthesized without microwave at different magnifications, (**d**) SAED pattern.

**Fig. 9:**
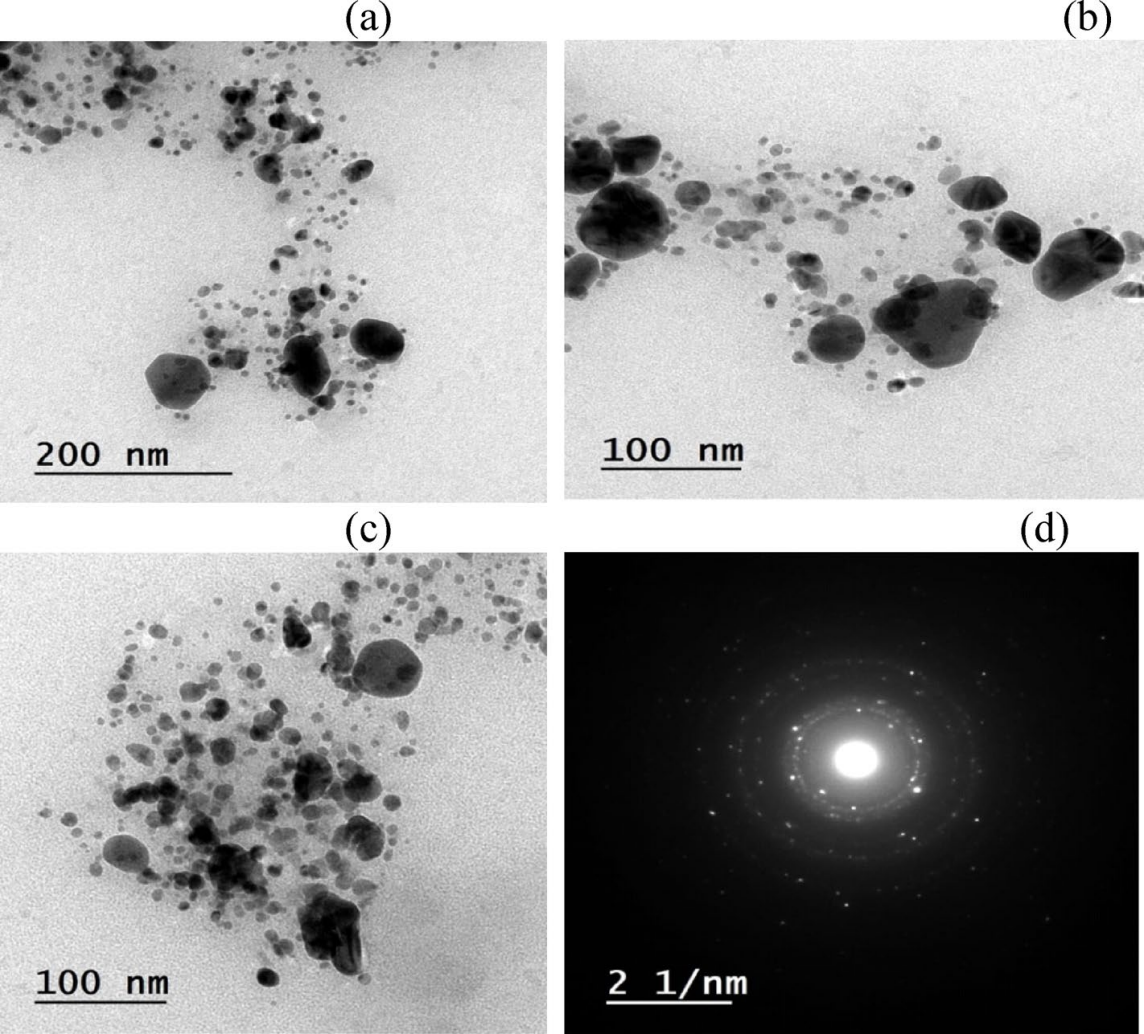
(**a**, **b** & **c**) TEM images of AgNP synthesized using microwave at different magnifications, (**d**) SAED pattern.

To confirm the shape, size, and morphology of the synthesized AgNPs, TEM analysis was performed. The TEM images (Figs. 8, 9) confirm that the AgNPs formed without and by microwave are in nanoscale, and the majority of the particles have spherical shape with an average diameter of 9–24 nm for that without microwave (Fig. 8) and 5–23 nm for that with microwave (Fig. 9). Both large and small particles were also observed. This result was as that during the microwave irradiation, smaller particles transformed into larger particles via subsequent crystallization, which required the nucleation and growth processes of larger particles from smaller ones. The results clearly show that the synthesized colloidal AgNPs were uniformly distributed, homogeneous, primarilyspherical, and well crystalline in nature, as shown in Figs. 8, 9. Because of the encapsulation of the AgNPs with the leaf extract of the *T. hamosa*, agglomeration of the AgNPs was not seen.

### Photocatalytic degradation of MB dye using AgNPs

**Fig. 10:**
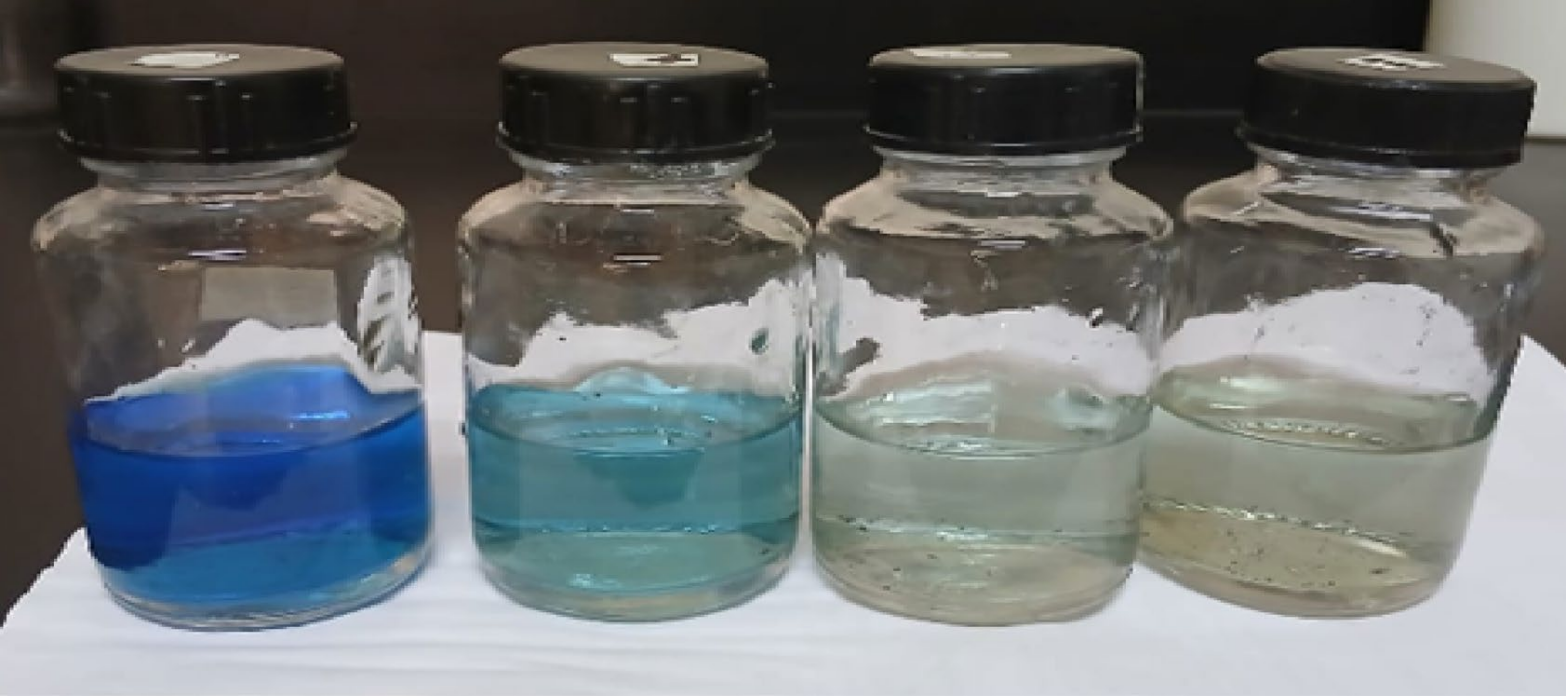
Photo degradation of MB dye using *Trigonella hamosa* leaf extract mediated synthesized silver nanoparticles.

By the decolonization of industrial dyes under sunlight and visible lamp irradiation, the photocatalytic activity of green-produced AgNPs was investigated. The photocatalytic performance of the synthesized AgNPs was evaluated using methylene blue dye, which displayed a major absorption band at 620 nm. It was observed that MB dye is converted from the oxidized form (deep blue) to the reduced form (leuco form), which is colorless in the presence of sunlight for 24 h with AgNPs synthesized from *Trigonella hamosa *L*.* (Fig. 10). The most probable explanation is that electrons are activated when photons strike the surface of the Ag NPs. The dissolved oxygen molecules from the solution receive the excited electrons from the surface of the AgNPs and change them into O_2_ radicals, which breakdown the dye and convert the organic color into simpler organic molecules^54^. Therefore, in the presence of visible lamp irradiation, the biosynthesized AgNPs may function as a stable and effective green catalyst for the degradation of MB.

MB dye is a cationic thiazine dye that has numerous applications in a several fields, including biology and chemistry. MB dye is used in the aquaculture sector as a chemotherapeutic and anti-malarial agent as well as indicator in analytical chemistry. Also, it is used to analyze the trace concentration of sulfide ions in water samples^55^.

The sun and visible lamp irradiation were irradiated to the MB dye solution, and the photocatalytic studies were conducted for 10 ppm MB dye concentration, 24 h sunlight irradiation, and 450 min under visible lamp irradiation.

In our study, it’s found that 96.2% degradation of MB occurs in the presence of 10 mg of AgNPs with an average size of 14 nm within 24 h under sunlight, and 94.9% within 7 h under a visible lamp. Table 3 shows the degradation of MB using different plant extract regarding to our study.

The proportion of dye degradation was calculated according to the following formula:

3 $$\% D = \left( {C_{o} - C} \right)/C_{o} * 100$$

where the degradation percentage is denoted by % D, C_o_ and C refer to the initial and final concentration of the MB dye after decolorization, respectively. The data of the degradation conditions (time and the rate constant values) are provided in Tables 4 and 5.

**Table 4:** Rate constants and kinetic studies for degradation of MB dye using AgNPs under solar light.

10 ppm Methylene Blue + 10 mg (AgNO_3_) + Solar light					
T (hr)	C	C/°C	Log C/°C	− Log C/°C	% D
0	10	1	0	0	0%
3	8.21	0.821	− 0.0856	0.0856	17.9
6	6.54	0.654	− 0.1844	0.1844	34.6
9	5.57	0.557	− 0.2541	0.2541	44.3
12	4.47	0.447	− 0.3496	0.3496	55.3
15	2.62	0.262	− 0.5816	0.5816	73.8
18	1.22	0.122	− 0.9136	0.9136	87.8
21	0.49	0.049	− 1.3098	1.3098	95.1
24	0.38	0.038	− 1.4202	1.4202	96.2

**Table 5:** Rate constants and kinetic studies for degradation of MB dye using AgNPs under visible lamp.

10 ppm Methylene Blue + 10 mg (AgNO_3_) + visible light					
T min	C	C/°C	Log C/°C	− Log C/°C	% D
0	10	1	0	0	0
30	9.67	0.967	− 0.0145	0.0145	3.3
60	9.03	0.903	− 0.0443	0.0443	9.7
90	7.9	0.790	− 0.1023	0.1023	21
120	6.89	0.689	− 0.1617	0.1617	31.1
150	5.33	0.533	− 0.2732	0.2732	46.7
180	4.61	0.461	− 0.3362	0.3362	53.9
210	3.74	0.374	− 0.4271	0.4271	62.6
240	3.07	0.307	− 0.5128	0.5128	69.3
270	2.6	0.260	− 0.5850	0.5850	74
300	1.99	0.199	− 0.7011	0.7011	80.1
330	1.58	0.158	− 0.8013	0.8013	84.2
360	1.16	0.116	− 0.9355	0.9355	88.4
390	0.7	0.07	− 1.1549	1.1549	93
420	0.62	0.062	− 1.2076	1.2076	93.8
450	0.51	0.051	− 1.2924	1.2924	94.9

**Fig. 11:**
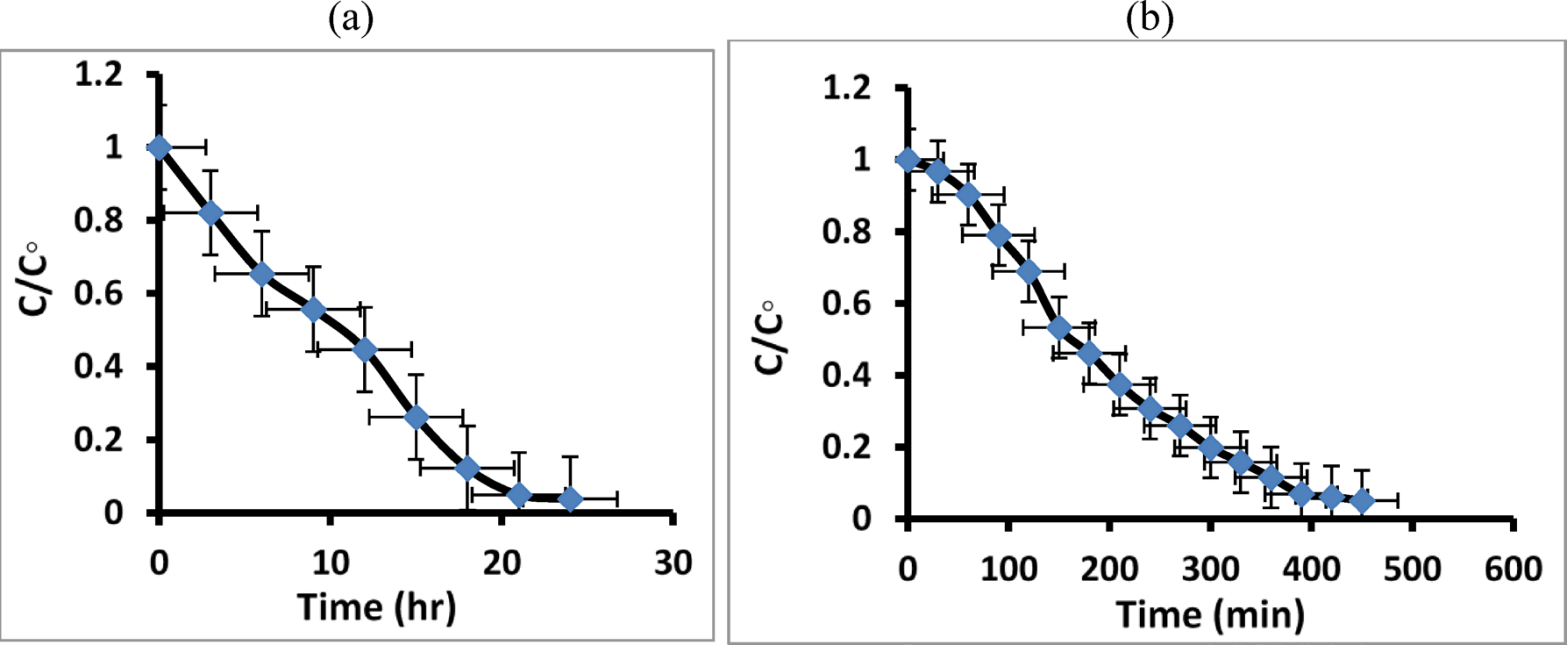
The relationship between (C/Co) and time (t) for MB dye degradation in maximum time 24 h under natural sunlight irradiation (**a**) and 450 min under visible lamp (**b**).

The plot of (C/C_o_) Vs time (t) (Fig. 11 a, b) confirms that the dye degradation increased with time and was maximum at 24 h under sunlight and 450 min under visible lamp irradiation. MB dye removal can occur when sunlight or visible lamp irradiation is present.

**Fig. 12:**
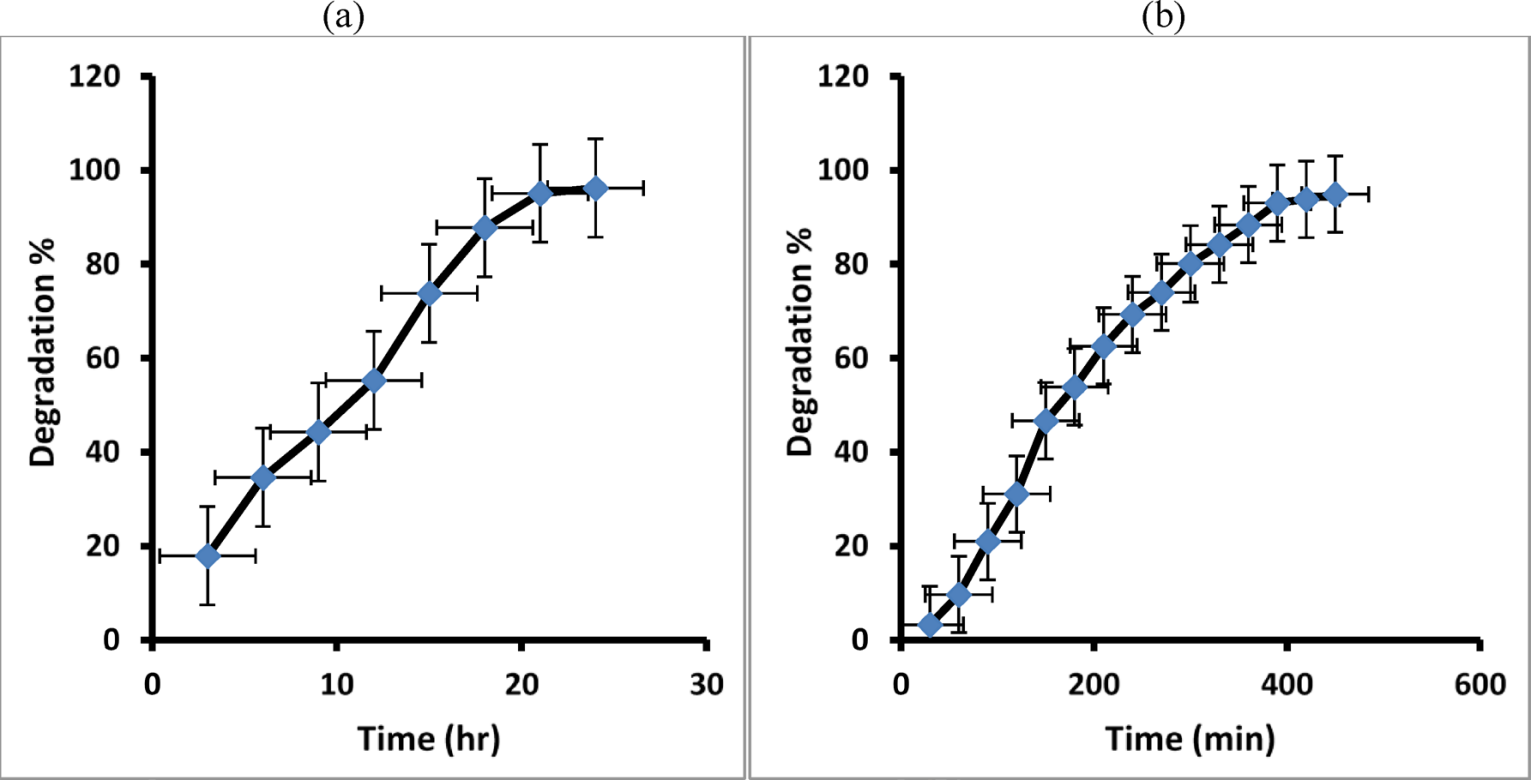
The degradation percentage of MB dye under natural sunlight irradiation (**a**) and under visible lamp (**b**).

In this work, we can estimate that 94.9% of the MB dye in distilled water is degraded using a visible lamp after 7 h, and around 96.2% is degraded after 24 h under natural sunlight irradiation, as shown in Fig. 12a, b.

### Photocatalytic degradation of paracetamol using AgNPs

The proportion of paracetamol degradation was calculated according to Eq. 3.

Where the degradation percentage is denoted by % D, C_o_ and C refer to the initial and residual concentration of the paracetamol solution after removal in equilibrium, respectively.

The specifics of the degradation in relation to time and the values of the rate constant.are provided in Tables 6 and 7.

**Table 6:** Rate constants and kinetic studies for AgNPs for paracetamol removal under solar light for 24 h.

50 ppm paracetamol + 10 mg (AgNO_3_) + Solar light					
T (hr)	C	C/°C	Log C/°C	− Log C/°C	% D
0	50	1	0	0	0
3	43.9	0.878	− 0.0565	0.0565	12.2
6	38.65	0.773	− 0.1118	0.1118	22.7
9	35.7	0.714	− 0.1463	0.1463	28.6
12	32.35	0.647	− 0.1890	0.1890	35.3
15	22.2	0.444	− 0.3526	0.3526	55.6
18	11.35	0.227	− 0.6439	0.6439	77.3
21	3.8	0.076	− 1.1191	1.1191	92.4
24	2.75	0.055	− 1.2596	1.2596	94.5

**Table 7:** Rate constants and kinetic studies of AgNPs for paracetamol removal under visible lamp for 7 h.

50 ppm paracetamol + 10 mg (AgNO_3_) + visible light					
T min	C	C/°C	Log C/°C	− Log C/°C	% D
0	50	1	0	0	0
30	49.85	0.997	− 0.0013	0.0013	0.3
60	48.15	0.963	− 0.0163	0.0163	3.7
90	47.95	0.959	− 0.0181	0.0181	4.1
120	46.75	0.935	− 0.0291	0.0291	6.5
150	46.55	0.931	− 0.0310	0.0310	6.9
180	46	0.92	− 0.0362	0.0362	8
210	44.25	0.885	− 0.0530	0.0530	11.5
240	37.7	0.754	− 0.1226	0.1226	24.6
270	34.95	0.699	− 0.1555	0.1555	30.1
300	27.7	0.554	− 0.2564	0.2564	44.6
330	19.6	0.392	− 0.4067	0.4067	60.8
360	13.4	0.268	− 0.5718	0.5718	73.2
390	5.05	0.101	− 0.9956	0.9956	89.9
420	4.2	0.084	− 1.0757	1.0757	91.6
450	3.95	0.079	− 1.1023	1.1023	92.1

**Fig. 13:**
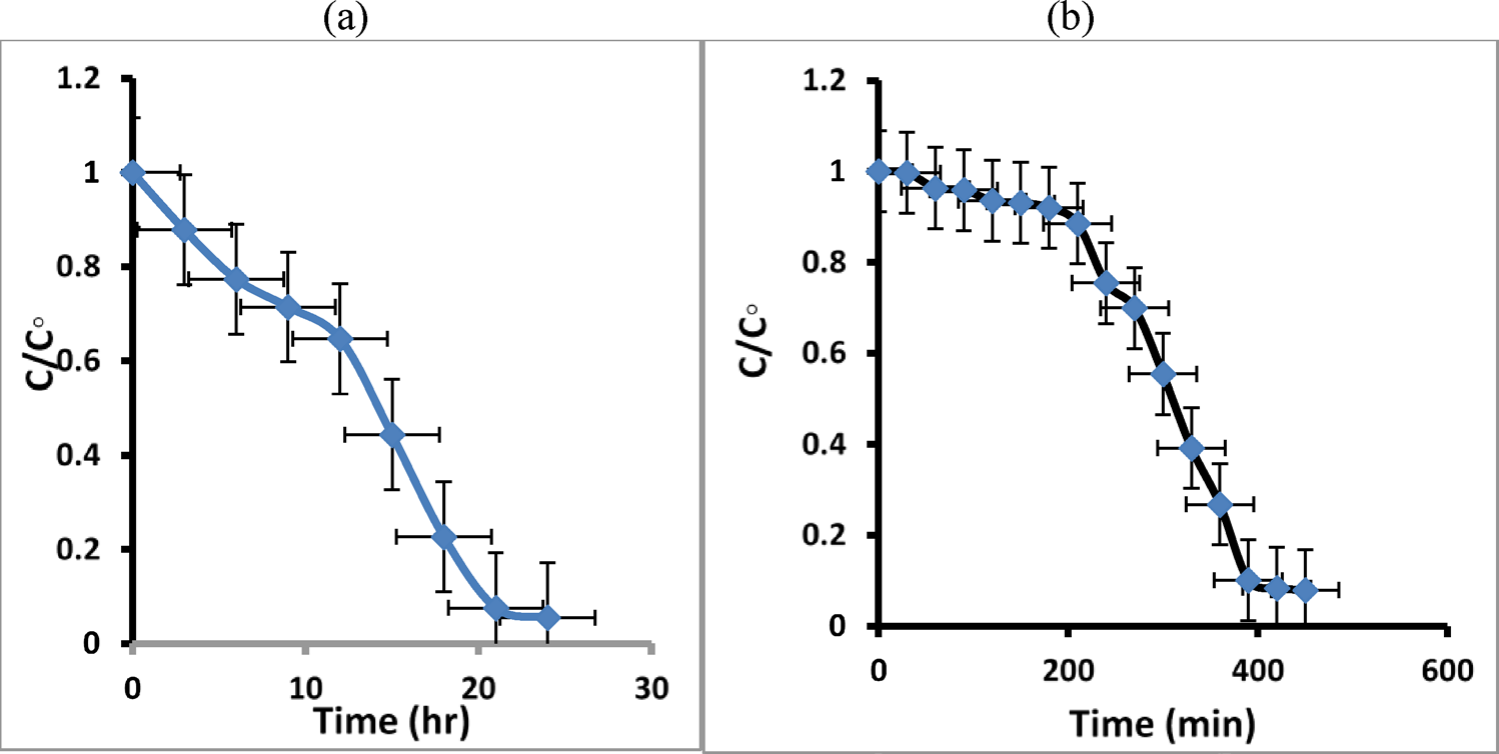
The relationship between (C/Co) and time (t) for paracetamol degradation in maximum time at 24 h under natural sunlight irradiation (**a**), and 450 min under visible lamp (**b**).

Figure 13a, b reveals the plot of (C/Co) vs. time (t), which confirms that the paracetamol degradation increased with time and was maximum at 24 h under sunlight and 450 min under visible lamp irradiation. Paracetamol removal can occur when sunlight or visible lamp irradiation is present.

**Fig. 14:**
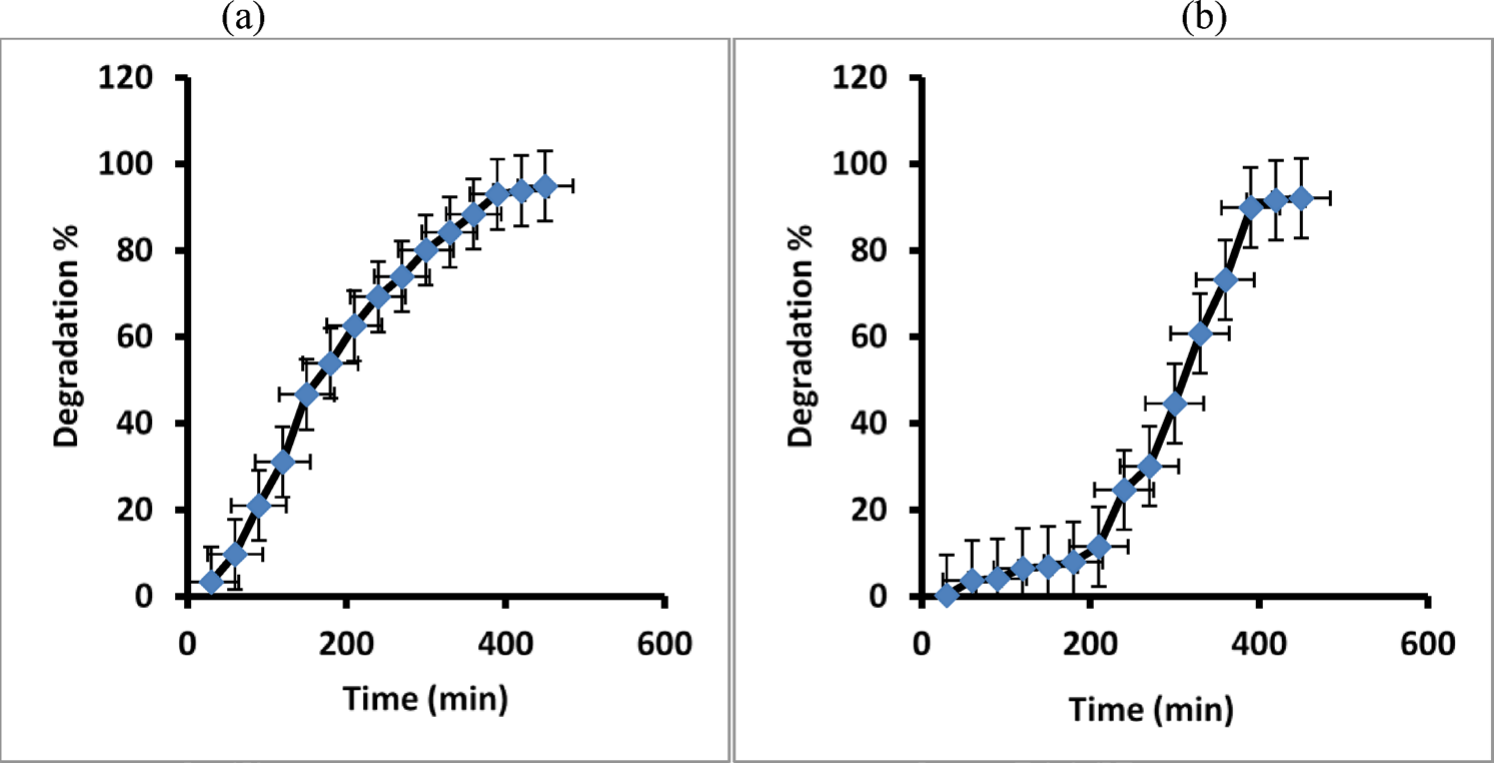
The degradation percentage of paracetamol under natural sunlight irradiation (**a**) and under visible lamp (**b**).

The resulted data in this work estimated that 92% of the paracetamol in distilled water can be degraded using visible lamp irradiation, and 94.5% is degraded after 24 h under natural sunlight irradiation, as shown in Fig. 14a, b.

### Effect of recycle times of AgNPs on the photo degradation of paracetamol and methylene blue dye under sun and visible lamp irradiation

A green photocatalysis technology has no concerns with waste disposal, thus it is vital to preserve AgNPs during each usage cycle.

**Fig. 15:**
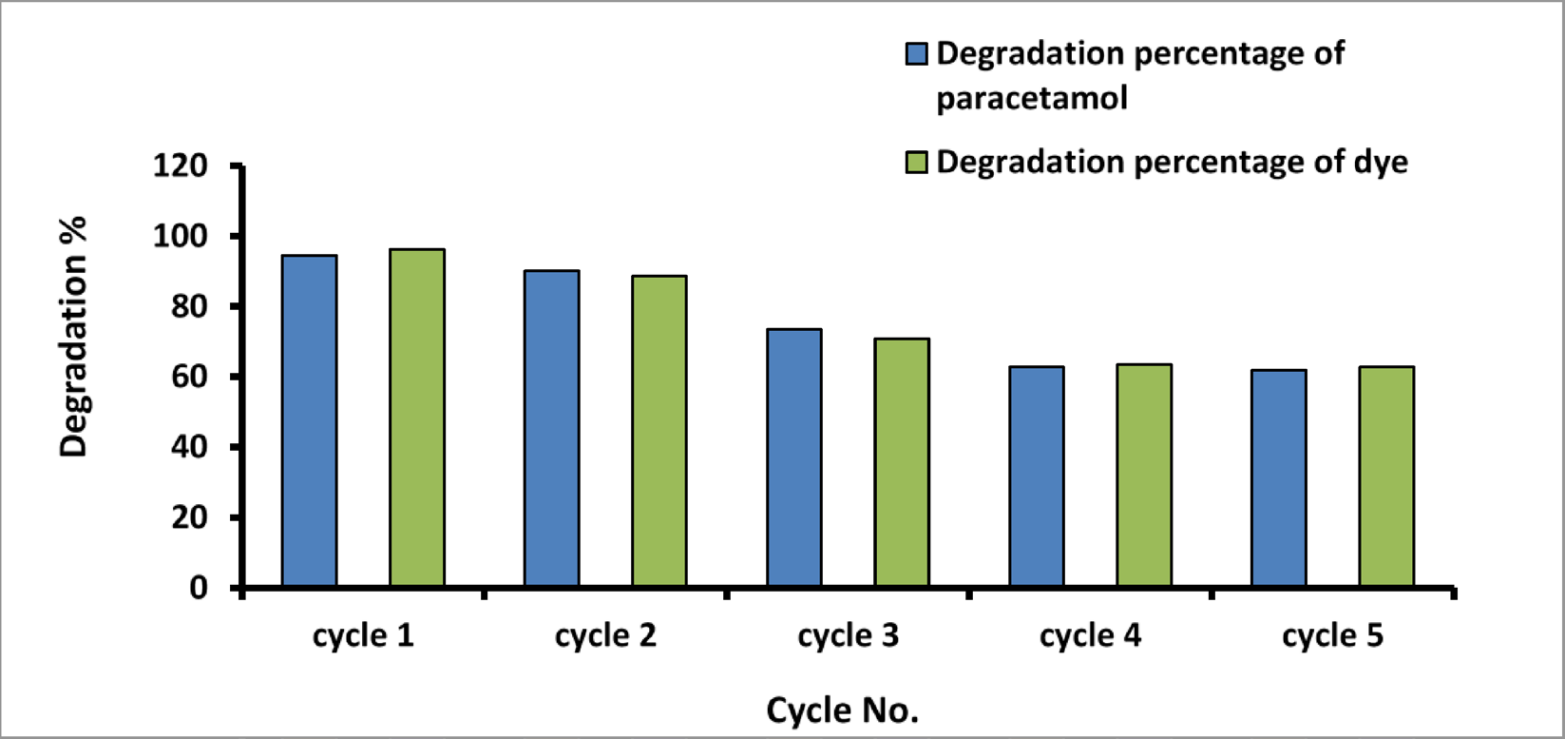
Effect of recycle times of on the photodegradation of paracetamol and MB dye under natural sunlight irradiation.

**Fig. 16:**
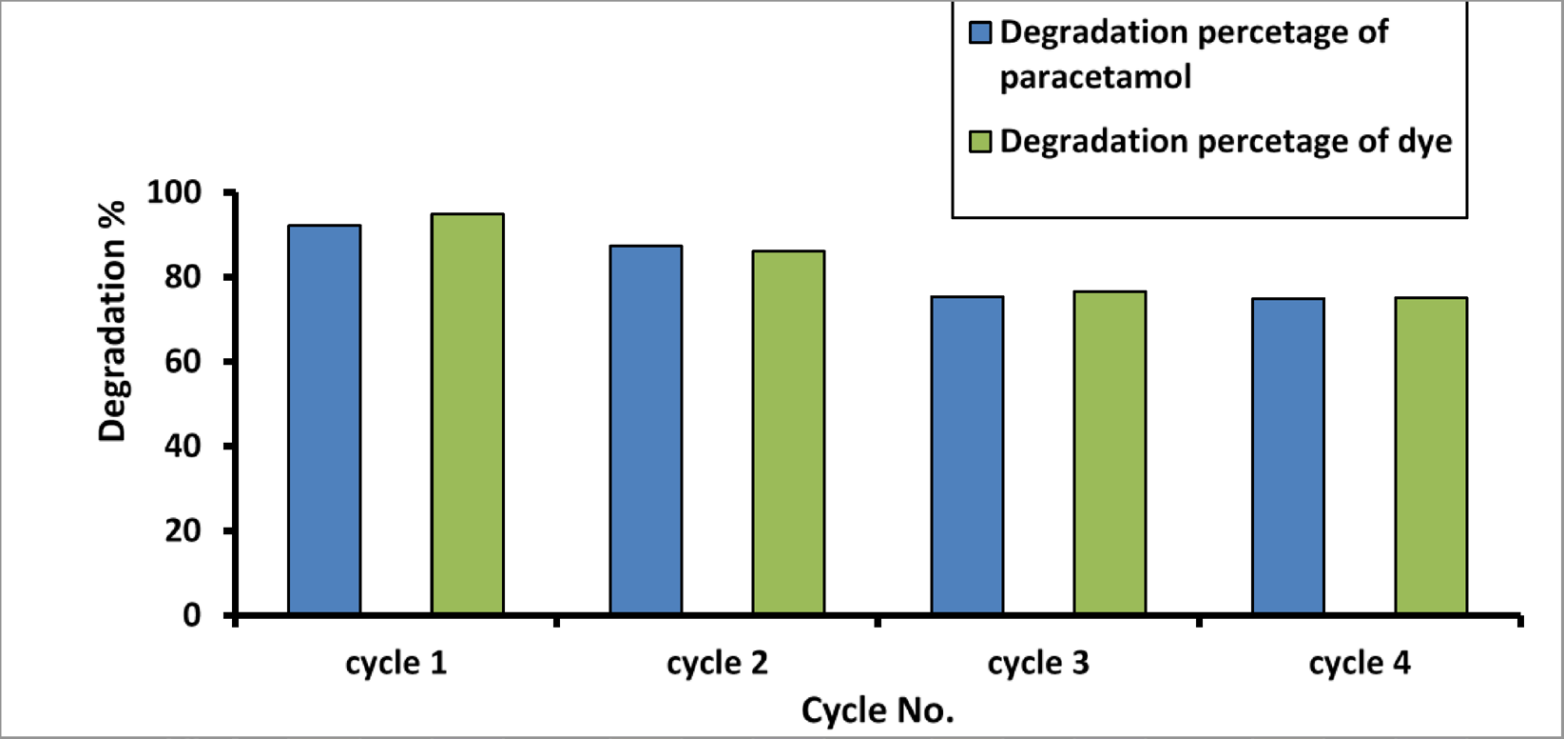
Effect of recycle times on the photodegradation of paracetamol and MB dye under visible lamp irradiation.

The reuse and regeneration of AgNPs are investigated, and the outcome is shown in Figs. 15 and 16. After each cycle, it is obvious that the photocatalyst was subjected to centrifugation, washing, and drying and then reused for subsequent degradation. The photodegradation of the Paracetamol and Methylene Blue under sunlight is reduced by about 30% after five cycles, and the photodegradation under visible lamp irradiation is reduced by about 20% after four cycles. The results confirm that AgNPs remain highly photocatalytically active after five cyclical experiments.

## Conclusions

In this work, AgNPs was synthesized using a microwave-assisted method technique as a novel approach, employing the leaf extract of *T. hamosa* as both a reducing and capping agent for the synthesis of nanoparticles in an aqueous solution. This method for synthesis of AgNPs is quick, simple, economical, and environmentally friendly. The nanoparticles exhibit stability in an aqueous medium for an extended period.

FTIR, XRD, TEM, and UV–vis methods were used to characterize the prepared AgNPs. The AgNPs yielded from this method were highly crystalline and well dispersed and found to be extremely effective catalysts for the degradation of MB dye and paracetamol drug.

Degradation of MB dye and paracetamol drug was performed using the prepared AgNPs under sunlight and visible lamp irradiation. The degradation of MB dye was found to be 96.2% and 94.9%, whereas that for paracetamol was 94.5% and 92% when subjected to sunlight and visible lamp irradiation, respectively. This novel method for green synthesis of AgNPs has various benefits and provides an efficient and cost-effective way to protect the environment.

Also, the most significant finding of the study is to use this green AgNPs for photocatalysis degradation of dyes and drugs.

## Data Availability

The datasets used and/or analysed during the current study available from the corresponding author (M.Nageeb Rashed, email: mnrashed@aswu.edu.eg) on reasonable request.
