# Correction: Microwave assisted green synthesis of silver nanoparticles using *Trigonella Hamosa L*. plant extract for the photodegradation of some water pollutants

**DOI:** 10.1038/s41598-026-35710-3

**Published:** 2026-02-02

**Authors:** M. Nageeb Rashed, Eman Abdelrady, Tereasa M. Ghabrial

**Affiliations:** 1 Department of Chemistry, Faculty of Science, Aswan University, Aswan, Egypt; 2 Unit of Environmental Studies and Development, Aswan University, Aswan, Egypt

Correction to: *Scientific Reports* 10.1038/s41598-025-21112-4, published online 27 October 2025

The original version of this Article contained errors. In Fig. 6, the labeling of the charts as (a) and (b) was incorrect.

**Fig. 6:**
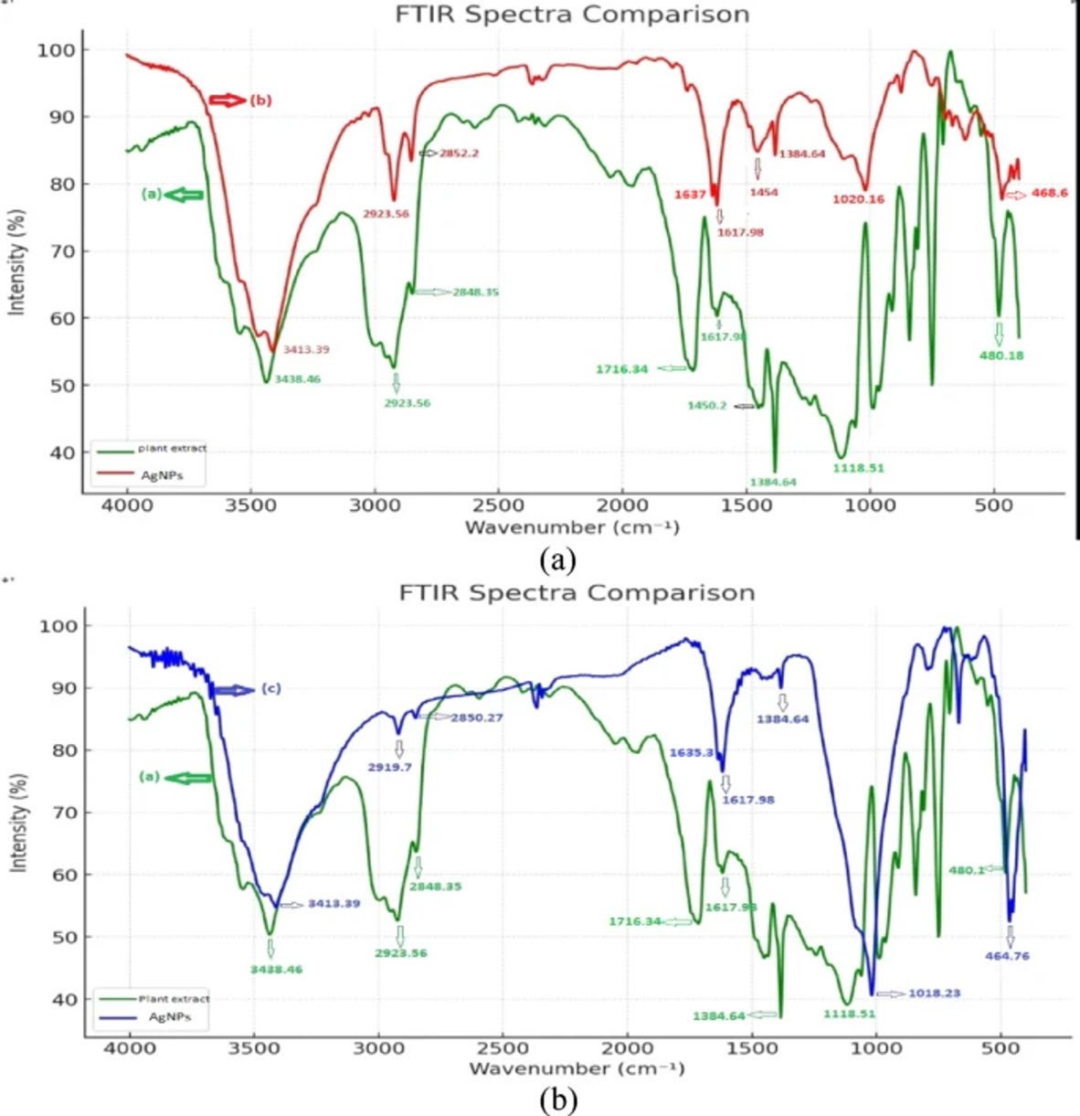
(**a**) FT-IR spectrum of an aqueous leaf extract, (**b**), and (**c**) synthesized AgNPs without and by microwave.

Furthermore, the legends of Figures 4, 5 and 16 contained errors, where:

Figure 4 legend: “Images of (**a**) mixture of plant extract and AgNO_3_ before microwave and (**b**) AgNP-after microwave.”

now reads:

“UV–vis absorption spectra of T.hamosa L leaves extract”.

Figure 5 legend “(a) UV–vis absorption spectra of AgNP synthesized with T. hamosa L. leaves extract, (a) without microwave irradiation (60 min stirring time, 70 °C), (b) under microwave irradiation after 5 min.recorded after 60 min under”.

now reads:

“UV–vis absorption spectra of AgNP synthesized with T.hamosa L leaves extract, a) without microwave irradiation (60 min stirring time, 70°C), b) under microwave irradiation after 5 min.”

Finally, Fig. 16 legend: “Effect of recycle times of on the photodegradation of paracetamol and MB dye under natural sunlight irradiation”.

now reads:

“Effect of recycle times on the photodegradation of paracetamol and MB dye under visible lamp irradiation.”

The original Article has been corrected.

